# Evaluation of Magnetization Transfer Contrast Sequences: Application to Monitor Age-Related Differences in Muscle Macromolecular Fraction

**DOI:** 10.3390/tomography11090103

**Published:** 2025-09-05

**Authors:** Austin Crispin-Smith, Ti Wu, Ilana R. Leppert, Agah Karakuzu, Shantanu Sinha, Usha Sinha

**Affiliations:** 1 Department of Physics, San Diego State University, San Diego, CA 92182, USA; austin.crispin-smith@roswellpark.org (A.-C.S.); twu3565@gmail.com (T.W.); 2 Brain Imaging Center, Montreal Neurological Institute, Montreal, QC H3A2B4, Canada; ilana.leppert@mcgill.ca; 3 NeuroPoly Lab, Polytechnique Montreal, Montreal, QC H3T1N8, Canada; agahkarakuzu@gmail.com; 4 Muscle Imaging and Modeling Laboratory, Department of Radiology, University of California San Diego, San Diego, CA 92037, USA; shsinha@health.ucsd.edu

**Keywords:** calf muscle magnetization transfer, aging muscle, quantitative magnetization transfer

## Abstract

Background/Objectives: Several sequences for magnetization transfer contrast (MTC) imaging are available, from indices of MTC ranging from quantitative magnetization transfer (qMT) that yields the macromolecular fraction to simple ratios of signal intensities with and without a magnetization transfer (MT) pulse. Aging muscle undergoes changes including an increase in fibrosis and adipose accompanied by fiber atrophy and loss. The objective is to evaluate five MTC sequences to study age-related differences in muscle tissue composition. Methods: The lower leg (calf) of 15 young (8M/7F, 25.8 ± 3.7 years) and 9 senior subjects (5F/4M, 68.4 ± 3.3 years) was imaged with the following sequences: multi-offset qMT fit to the Ramani and Yarnykh models, single-offset qMT two-parameter fit to the Ramani model, a semi-quantitative *MT_sat_* sequence, magnetization transfer ratio (*MTR*), and MTR-corrected (*MTR_corr_*) for B1 inhomogeneities. *T1* mapping was also performed. Statistical analysis was performed to identify significant age-related and regional (intermuscular) differences. Results: Significant age-related decreases (*p* < 0.001) in macromolecular fraction (from two-parameter fit), *MT_sat_*, *MTR*, and *MTR_corr_* were identified. A significant age-related increase in *T1* (*p* < 0.001) was also identified. Pearson correlation coefficients between *T1* and MTC indices were weak to moderate but significant. Conclusions: Age-related decreases in MTC may reflect that loss of myofibrillar proteins dominates the increase in collagen content with age. Further, the modest correlation of MTC indices with *T1* indicates that all the age-related differences in MTC cannot be explained by an increase in inflammation. The *MT_sat_* sequence was identified as the most clinically relevant in terms of acquisition speed, post-processing simplicity, and ability to identify age-related differences in macromolecular fractions.

## 1. Introduction

The age-related loss of muscle mass accompanied by a disproportionate loss in muscle force is well-established [1]. The loss in muscle force arises from age-related remodeling in muscle and neural factors, and as more recently reported, in the extracellular matrix (ECM) [2,3]. Age-related changes in muscle-related factors include muscle atrophy leading to a decrease in fiber size and fiber density and concomitant decrease in myofibrillar proteins. The remodeling of the ECM includes an increase in intramuscular connective tissue as well as in collagen content [4,5]. Non-invasive mapping of the macromolecular fraction could potentially provide a better understanding of the aging process, and further, inform therapeutic/physical rehabilitation strategies to mitigate the effects of the remodeling of muscle fibers and/or ECM [6].

Macromolecules are not directly observed in traditional magnetic resonance imaging (MRI) due to the very short T2 of the protons bound to macromolecules [7]. However, magnetization transfer contrast (MTC) imaging enables an indirect evaluation of the macromolecular content by selective saturation of bound protons [7,8]. Recently, MTC imaging has been applied to skeletal muscles including calf and thigh muscles [7,9,10,11]. There are several approaches to MTC imaging, ranging from quantitative magnetization transfer (qMT) with fitting to a two-pool model [12,13], to a simple ratio of image intensities acquired with and without the magnetization transfer pulse, called the magnetization transfer ratio (*MTR*) [14]. Sinclair et al. (2010) [9] applied the two-pool exchange model to multi-offset data to derive the macromolecular fraction of calf muscles; the study showed the feasibility of qMT of skeletal muscle. A version of qMT that is based on ultrashort TE imaging, called UTE-MT, has also been successfully applied to extract the calf muscle macromolecular fraction [15]. qMT studies have also been performed on thigh muscle using the multi-offset qMT protocol and a faster version using single-offset protocols [10,11]. This latter fast alternative requires only two images but still fits to the two-pool model, making reasonable assumptions based on empirical observations about the values of many parameters of the model [10]. The single-offset protocol appears promising as a fast, clinically feasible technique and was shown to have less variance compared to the longer multi-offset qMT protocols. While *MTR* is a simple approach involving no complex post-processing, it is sensitive to sequence details as well as to B1 inhomogeneities and to *T1*; this approach has been modified to incorporate a B1-corrected magnetization transfer ratio (*MTR_corr_*) [16]. A semi-quantitative index of magnetization transfer (MT), *MT_sat_*, has also been applied to studying skeletal muscle, and this sequence has an inherent correction for RF inhomogeneity and *T1* relaxation, the latter two are confounding factors for *MTR* [7,17,18].

As detailed above, there are several techniques to determine MTC, ranging from ratios of signal intensities to computation of the macromolecular fraction. These different techniques are a tradeoff between speed and quantitative estimate of the macromolecular fraction. In studying the aging muscle, their ultimate utility will be determined by their effectiveness as a direct biomarker or surrogate biomarker of macromolecular changes/differences with age in clinically feasible acquisition times. The focus of this paper is to evaluate five magnetization transfer contrast sequences (multi-offset qMT, single-offset qMT, *MT_sat_*, *MTR*, and *MTR_corr_*) to detect age-related differences in macromolecular fraction between young and senior cohorts using a prospective cross-sectional study design.

## 2. Materials and Methods

### 2.1. Phantom

Validation of the different MT sequences was performed using agarose phantoms that were fabricated in-house with known macromolecular fractions. The theoretical macromolecular fraction was estimated as the fraction of protons, *f*, contained within the bound pool to the total number of protons for each agarose concentration. The phantoms used in this study were synthesized from nickel nitrate-doped agarose following the work of Christoffersson et al. (1991) [19]. The gel phantom was selected due to the simplicity of preparation, relatively long shelf life, and flexibility to mimic different soft tissue by varying *T1* and T2 relaxation times. Gel phantoms were synthesized at agarose concentrations of 0.5, 1, 2, 3, and 4%. The proposed pulse sequences were evaluated with the agarose gel phantoms with a known macromolecular fraction.

### 2.2. Human Subjects

The cohort consisted of 15 young (8 male, 25.8 ± 3.7 years) and 9 senior subjects (5 male, 68.4 ± 3.3 years). Subjects were included in this study after written informed consent had been obtained. This study was carried out under the approval of the Institutional Review Board of San Diego State University, San Diego, and the studies were conducted in accordance with the local legislation and institutional requirements. All the studies were conducted on a 3 T clinical MR scanner (MAGNETOM Prisma, Siemens Healthineers, Forchheim, Germany). Subjects were positioned feet first, supine in the scanner. The lower right leg was positioned on the spine coil (12 channels activated) while a 4-channel flex coil was wrapped around the anterior part of the lower leg.

### 2.3. Pulse Sequences

Muti-offset qMT: Fifteen volumes were acquired using 3D FLASH sequences for the multi-offset qMT with the following parameters: TE/TR/FA: 3 ms/50 ms/6°, matrix: 128 × 128 × 16 (voxel size:1.56 mm × 1.56 mm × 10 mm), FOV:20 cm × 20 cm, GRAPPA factor: 2. Fat suppression was accomplished using a water excitation pulse (fast, 1:1 binomial composite pulse). Sequences were acquired with the MT pulse at two flip angles: 350° and 500° with 7 offsets each: 1, 2, 5, 10, 20, 50, and 100 kHz. A Gaussian RF pulse with 200 Hz bandwidth and 10.24 ms pulse duration was used for the MT pulse. MT pulses were applied during a preparation time of 5 s before the start of acquisition with a TR of 50 ms; the TR matched that of the acquisition sequence. This resulted in a total of 100 (≡5000/50) MT pulses with a duty cycle of 24%.

An additional volume was acquired with no MT preparation and used for normalization. The total time for all scans for multi-offset qMT was 18 min 30 s.

Single-offset qMT and MTR: Two volumes were acquired with a 3D FLASH sequence for both the single-offset qMT and *MTR* analysis: TE/TR/FA: 3 ms/50 ms/6°, matrix: 128 × 128 × 16 (voxel size: 1.56 mm × 1.56 mm × 10 mm), FOV: 20 cm × 20 cm, GRAPPA factor: 2. Fat suppression was accomplished using a water excitation pulse (fast, 1:1 binomial composite pulse). Sequences were acquired with an MT pulse of flip angle 650° and offsets 1 and 100 kHz; other parameters of the MT pulse as well as the MT preparation pulses were the same as specified for the multi-offset qMT. The 100 kHz image was used for normalization. The total time for the single-offset qMT and *MTR* was 2 min 28 s.

MT_sat_: Three 3D FLASH volumes were acquired with the following parameters: TE/TR/FA: 3 ms/50 ms/4° (proton density weighted, PDw), 10° (with MT pulse, MTw), 20° (T1 weighted, *T1w*), matrix: 128 × 128 × 16 (voxel size 1.563 × 1.563 × 10 mm). Fat suppression was accomplished with the water excitation (fast, 1:1 binomial composite pulse) pulse. The magnetization transfer pulse was a Gaussian RF pulse, 375 Hz bandwidth, 9.984 ms duration, 1.2 kHz offset, 500° flip angle. The total time for the three scans for *MT_sat_* was 3 min 12 s.

T1 mapping (Variable Flip Angle (VFA)): *T1* maps were derived from 3D FLASH sequences (TE/TR: 3 ms/25 ms) acquired at three flip angles (5°, 15°, 25°) with geometry matching the sequences listed above. Fat suppression was accomplished using a water excitation pulse (fast, 1:1 binomial composite pulse). The total time for the three scans was 1 min 45 s.

B1 correction: We acquired a 2D turboFLASH sequence (TE/TR: 1.95 ms/18,040 ms) with geometric factors matching the qMT and the VFA sequences to compute B1 maps. The sequence does not allow contiguous slices, so two passes of the sequence with a 100% slice thickness gap were acquired to obtain the same coverage as the 3D sequences. Fat suppression was accomplished by fat saturation. The B1 maps were used to correct the flip angles of the VFA *T1* sequence, the MT RF pulse FA of the qMT sequences, and the flip angles (read) of the *MT_sat_* sequences. The time for the B1 correction maps for the two passes of the sequence was 1 min 16 s.

B0 correction: This was performed using a 3D dual echo FLASH sequence (TE1/TE2/TR: 4.92 ms/7.38 ms/320 ms) with geometry parameters matching the other sequences. Fat suppression was not available in the B0 correction sequence. The phase maps at the two echoes were processed to yield the B0 maps. The time for the B0 correction map was 1 min 24 s.

### 2.4. Image Analysis

Pre-processing: Prior to processing the data, the image volumes were all affine registered to the reference volume (chosen as either the volume acquired with no MT pulse or with the large RF offset) in *fsl* [20]. Affine registration (*fsl* function: FLIRT) is a linear registration that translates, rotates, zooms, and shears one image to match it with another. These transformations were sufficient to correct for the calf muscle motion between scans including small shape changes that may occur as the muscle is deformable. Images were examined manually to confirm that the registration resulted in a better alignment across scans and did not introduce any gross errors. The registered volumes were then denoised using SUSAN in *fsl* [21]. B0 maps were also calculated in *fsl* (https://fsl.fmrib.ox.ac.uk/fsl/docs/#/registration/fugue?id=fugue, accessed on 1 March 2022). *T1*, *MT_sat_*, and the multi-offset, two-pool Ramani and Yarnkyh models were fit using *qMRLab* software from NeuroPoly Lab (https://github.com/neuropoly/qMRLab/releases, accessed on 1 March 2022), Release v2.4.1. The single-offset data and *MTR* analysis were performed using software developed in-house. The B1 maps were used to correct the excitation RF (read) pulse flip angle for the *T1*, *MT_sat_* analysis and for the MT RF pulse flip angle in the qMT analysis. The B0 maps were used in correcting the frequency offsets for the qMT analysis. *T1* maps were used in the multi-offset and single-offset *qMT* analysis for the longitudinal relaxivity of the free pool.

Muti-offset qMT: Analysis was performed based on the ‘two-pool’ model proposed by Ramani et al. (2002) [13] using the implementation by Cabana et al. (2015) [22] and the associated software, *qMRLab*. The signal following saturation by the MT pulse may be derived from the Bloch equation and expressed as a function of eight fundamental parameters: $S_{0}$, R_A_, R_B_, $T_{2}^{A}$, $T_{2}^{B}$, $M_{0}^{A}$, F, and R.

$$S_{qMT}(\omega_{1},\Delta f)=\frac{S_{0}(R_{B}[\frac{RM_{0}^{A}F}{R_{A}}]+R_{B}+R_{RFB}+RM_{0}^{A})}{\left[\frac{RM_{0}^{A}F}{R_{A}}](R_{B}+R_{RFB})+(1+[\frac{\omega_{1CWPE}}{2\pi \Delta f}{]}^{2}[\frac{1}{R_{A}T_{2}^{A}}])(R_{RFB}+R_{B}+RM_{0}^{A}\right)}$$

where *S*_0_ is the observed signal intensity in the absence of the MT pulse, and S_qMT_(ω_1_, *Δf*) is the observed signal at a frequency offset, *Δf* and at a flip angle, $\omega_{1}$. The subscript A refers to the free pool, and B the bound pool, R_A_ is the longitudinal recovery rate of the free pool, R_B_ is the longitudinal recovery rate of the bound pool, R is rate constant describing the magnetization exchange between the two pools, F is the pool size ratio expressed as the ratio of the equilibrium magnetization of the bound pool to the free pool, M0BM0A, and R_RFB_ is the absorption line shape of the bound pool, which is modeled as a super-Lorentzian function. The qMT image volumes were normalized to the volume acquired with no MT pulse. *R_B_*, relaxivity of the bound pool, was fixed at 1 s^−1^ since several studies have shown that this parameter has little effect on the other fitted parameters [9]. R_A_, relaxivity of the free pool, was constrained by the relaxivity measured from the *T1* mapping sequence. The ‘two-pool’ model is derived assuming continuous excitation, whereas implementations on clinical scanners use pulsed excitation. To account for this, the RF MT pulse is approximated, and the two models considered here are the Ramani and the Yarnykh models [13,23]. In the Ramani model, the approximation of the RF pulse, $\omega_{1mt},$ to the equivalent continuous wave RF, $\omega_{1CW},$ is given by [13]

$$\omega_{1CW}^{2}=\frac{1}{TR}\int_{0}^{t_{mt}}\omega_{1mt}^{2}(t)dt$$

Here, TR is the pulse repetition time while t_mt_ is the duration of the magnetization transfer pulse. In the Yarnykh model, the approximation of the RF pulse, $\omega_{1mt},$ to the equivalent continuous wave RF, $\omega_{1CW},$ is given by [23]

$$\omega_{1rms}^{2}=\frac{1}{t_{mt}}\int_{0}^{t_{mt}}\omega_{1mt}^{2}(t)dt$$

The fit of the normalized model yields the following tissue micro-structural parameters: F, the pool size ratio; $RM_{0}^{A}$, the rate of magnetization transfer from the bound to the free pool; and $T_{2}^{A}$ and $T_{2}^{B}$, the T2 of the free and bound pools, respectively. $S_{0}$ is not an output of the fit since the normalized signal (normalized to the image with no MT pulse) is used as the input. A related index, *f*, the macromolecular fraction, is computed as M0B(M0A+M0B), and this index is reported in the rest of the current paper.

Single-offset qMT: The two-parameter qMT approach was initially proposed for brain studies based on observations that three parameters of the two-pool model, $RM_{0}^{A}$, $T_{2}^{B},$ and $\frac{1}{R_{A}T_{2}^{A}}$, have small variation across pathophysiological changes or across different tissues [24]. Hence, in the two-parameter qMT approach, these ‘non-varying’ parameters are fixed, and the reference image is generated using a large-frequency offset MT pulse. The latter approach is possible since a saturation pulse sufficiently off-resonance does not affect the bound pool; thus, there is no saturation effect on the free pool, and it can serve as the reference image without MT effects. The two-parameter approach was also successfully extended to thigh muscle imaging [10]. Following the latter work, the following parameters were kept constant in the current paper: $T_{2}^{B}$ = 6 µs, $R^{A}T_{2}^{A}$ = 0.025, $RM_{0}^{A}$ = 48 [10]. The 2-parameter model uses the Ramani approximation to the MT RF pulse. As in the multi-offset qMT model, $S_{0}$, is not fitted as normalized signal intensities are used. After normalization, F may be solved directly from the equation below ($S_{n}$ is the normalized observed signal at the lower-frequency offset). $R_{A}$ is derived from the measured longitudinal relaxivity of the free pool.

$$\frac{F}{R_{A}}=\frac{\left(R_{RFB}+R_{B}+RM_{0}^{A})(1-S_{n}(1+[\frac{\omega_{1CWPE}}{2\pi \Delta f}{]}^{2}\frac{1}{R_{A}T_{2}^{A}})\right)}{RM_{0}^{A}(S_{n}(R_{RFB}+R_{B})-R_{B})}$$

MT_sat_: *MT_sat_* is the percentage saturation imposed by one MT pulse during TR. This parameter is obtained by a linear transformation of the inverse signal, using two reference experiments of proton density (PD_w_) and *T1*-weighting (*T1*_w_) in addition to an MT-weighted (MT_w_) sequence. *MT_sa_*_t_ is a phenomenological quantity and is largely independent of excitation flip angle and longitudinal relaxation. *MT_sat_* is solved from the following expression and is represented by δ [7,17]:

$$S_{mt}=A\alpha \frac{R_{1}TR}{\frac{\alpha^{2}}{2}+\delta +R_{1}TR}$$

where *S_mt_* is the signal in the MT weighted sequence, *A* is the amplitude of the echo at *TE*, *R*_1_ is the longitudinal relaxivity, α is the flip angle, and δ is the percentage saturation imposed by one MT pulse, *MT_sat_*. The parameters *A* and *R*_1_ are estimated from the PD_w_ and *T1*_w_ sequences, respectively. The *qMRLab* software includes the corrected equations in erratum for [17], and further, offers an empirical correction for B1; this option was included in the calculation of *MT_sat_*. *MT_sat_* is independent of pulse sequence, *T1*, and to the first order, to B_1+_ (RF transmits field inhomogeneities). In addition, minor higher-order RF dependencies are corrected for with the following semi-empirical equation [25]:

$$\delta_{corrected}=\frac{\delta_{uncorrected}(1-0.4)}{1-0.4B_{1+}}$$

where $\delta_{uncorrected}$ is the original MT value, and B_1+_ is the relative flip angle with respect to the nominal flip angle. In this equation, 0.4 is chosen as a B1 correction factor.

MTR and MTR corrected (MTR_corr_): The simplest imaging technique to obtain an estimate of the MT effect is the magnetization transfer ratio (*MTR*) calculated from the signal intensity with and without the off-resonance MT RF pulse. Since it requires only two measurements, it is fast and clinically practical. However, *MTR* values are dependent on pulse sequence, *T1*, B_1+_, and MT pulse frequency offset. Ropele et al. developed an MTR correction method by assuming an idealized linear relationship between MTR_error_ and B1_error_ [26]. The method first solves for the linear factor ‘*k_specific_*’ for a specific tissue group, then extrapolates the relation to all tissue groups by the following method. In the current study, the correction used data from the tibialis posterior muscle in all acquired slices to solve first for the linear factor and then to derive an independent tissue ‘*k*’ value, as detailed below (each subject was processed individually).

$${MTR}_{measured}={MTR}_{corr}+k_{specific}B_{1error}\;k_{specific}=k{MTR}_{corr}\;{MTR}_{corr}=\frac{{MTR}_{measured}}{kB_{1error}+1}$$

**Figure 1:**
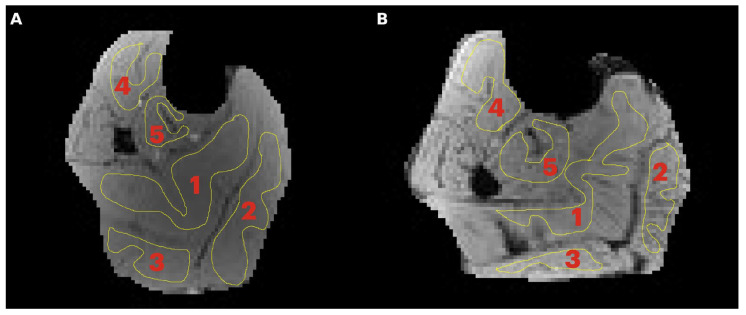
Transverse image of the lower leg of a young (**A**) and senior (**B**) subject with typical regions of interest manually contoured in the soleus (1), medial gastrocnemius, MG (2), lateral gastrocnemius, LG (3), tibialis anterior, TA (4), and tibialis posterior, TP (5). Care was taken to limit the ROI to the inside of the muscle borders and to avoid major fascicles.

Region of interest analysis: A region of interest was manually positioned in each of five calf muscles: medial gastrocnemius (MG), lateral gastrocnemius (LG), soleus (SOL), tibialis posterior (TP), and tibialis anterior (TA) (Figure 1). Care was taken to avoid low- and high-intensity regions within muscle as well as the edges of the muscle. Low-intensity regions were either fat (since the images were acquired with fat suppressed) or fascicles (connective tissue) while high-intensity areas potentially correspond to blood vessels. Parametric images were computed for all the MT indices evaluated in the current paper; however, the quantitative indices were computed from the average signal intensity in the muscle ROIs (Figure 1) extracted from the acquired images.

Statistical analysis: The outputs of the different MTC methods are *f*, the macromolecular fraction from multi-offset qMT, *f* from single-offset qMT, *MT_sat_*, *MTR*, and *MTR_corr_*. In addition, age-related differences in *T1* between the young and old cohorts were statistically analyzed. Data was analyzed for normality using Q-Q plots and the Shapiro–Wilk statistic. *T1* data was normally distributed; differences in *T1* between age groups and intramuscular regions as well as potential interaction effects were assessed using two-way factorial ANOVAs (age × region). Here, Levene’s test was used to test the assumption of homogeneity of variance and, in case of significant ANOVA results for the factor ‘region’, Bonferroni-adjusted independent sample t-tests were used for post hoc analyses. The magnetization transfer indices were not distributed normally (Shapiro–Wilk test, *p* < 0.05). In the absence of a non-parametric alternative to a factorial ANOVA, differences between age groups and muscles for these parameters were independently tested with Mann–Whitney U and Kruskal–Wallis tests, respectively. For the latter, Bonferroni-adjusted U-tests were also used for post hoc analyses. Further, for comparisons between age groups, effect sizes were expressed as the Hodges–Lehmann estimator of the median difference (raw effect size), with accompanying 95% confidence intervals and as the rank-biserial correlation, r (standardized effect size). Data are reported as mean ± SD for the variables that are normally distributed (*T1*), and median ± interquartile range (IQR) for those outcome variables that are not normally distributed (all magnetization transfer indices including *f*). IQR is a descriptive statistic for the spread of the data and is equal to the difference between the upper and lower quartiles, and the median is the corresponding measure of central tendency. Linear regression between *T1* and each of the MTC parameters was also performed to determine the correlation between magnetization transfer contrast indices and *T1*. For all tests, the level of significance was set at α = 0.05 (two-tailed). The statistical analyses were carried out using SPSS for Mac OSX (SPSS 21.0, SPSS Inc., Chicago, IL, USA).

## 3. Results

### 3.1. Agarose Phantom Validation

Table 1 lists the value of *f*, the macromolecular fraction of the agarose gel phantom extracted from the three quantitative MT sequences as well as the theoretical *f* values calculated from the concentration of agarose. The *f* value calculated from the multi-offset, Ramani model (*f(Ram*)) yielded values closest to the theoretical value of the macromolecular fraction for each concentration of agarose gel. The multi-offset, Yarnykh model (*f(Yar)*) yielded higher values than the calculated values. The single-offset fit (*f(so)*) is derived from the Ramani model, and since the three fixed parameters of the model changed with agarose concentration, the appropriate values of the fixed parameters from the Ramani model at each concentration were used in computing *f* at each concentration.

**Figure 2:**
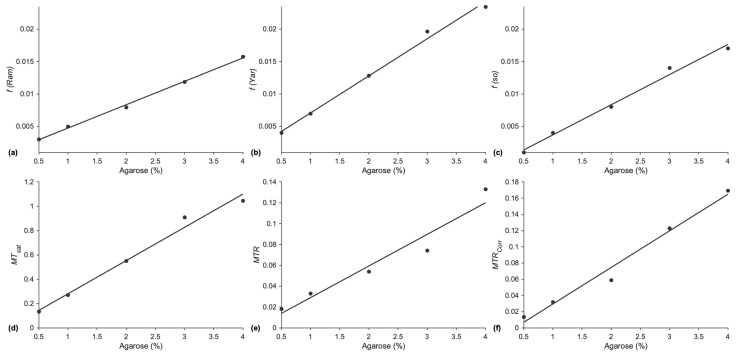
Macromolecular fractions and MTC indices as a function of % agar concentration (dilled circles are data points, the solid line is a linear fit). (**a**) *f* from multi-offset data fits to the Ramani model, *f(Ram)* (R^2^: 0.998, *p* = 5.4 × 10^−5^), (**b**) *f* from multi-offset data fits to the Yarnykh model, *f(Yar)* (R^2^: 0.993, *p* = 2.5 × 10^−4^), (**c**) *f* from single-offset data *f(so)* (R^2^: 0.990, *p* = 4.2 × 10^−4^), (**d**) *MT_sat_* (R^2^: 0.984, *p* = 8.8 × 10^−4^), (**e**) *MTR* (R^2^:0.941, *p* = 6.2 × 10^−3^), and (**f**) *MTR_corr_* (R^2^: 0.981, *p* = 1.1 × 10^−3^).

The other MTC indices are surrogate markers of *f*, and thus, only the linearity with agar concentration was evaluated (Figure 2). The fits of the MTC parameters to agarose concentration are linear, with the highest R^2^ values/lowest *p*-values for *f(Ram)* and lowest R^2^/highest *p* values for *MTR*. The effect of the B1 correction for *MTR* is seen in the increased R^2^ values for the linear fit and lower *p*-values of *MTR_corr_* compared to the uncorrected *MTR*. The calibration data show that these indices can be used to monitor differences/changes in the macromolecular fraction.

### 3.2. *f* and MTC Indices in Young and Old Cohorts

**Figure 3:**
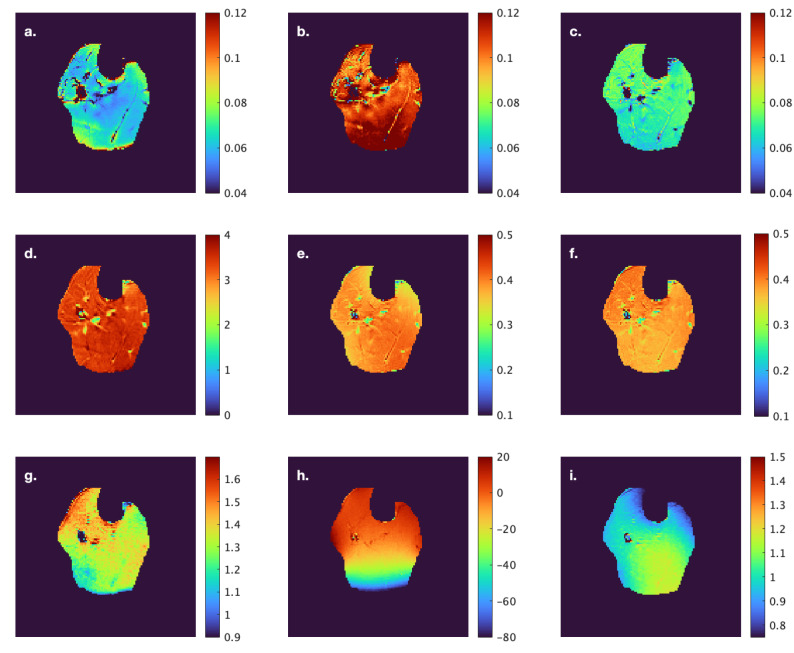
Computed *f* and *MTC* indices at one anatomical location from a subject from the young cohort. *f(Ram)* (**a**), *f(Yar)* (**b**), *f(so)* (**c**), *MT_sat_* (**d**), *MTR* (**e**), *MTR_corr_* (**f**), *T1* (**g**), B1 (**h**), and B0map (**i**). The images are displayed with a colormap to facilitate visualization of differences across muscles. The corresponding anatomical image with labeled ROIs is shown in Figure 1A.

**Figure 4:**
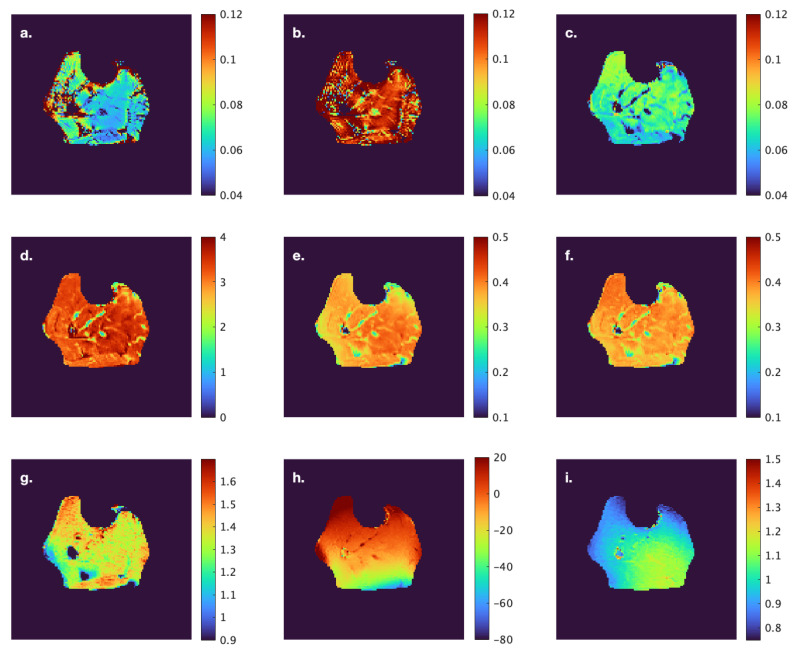
Computed *f* and *MTC* indices at one anatomical location from a subject from the senior cohort. *f(Ram)* (**a**), *f(Yar)* (**b**), *f(so)* (**c**), *MT_sat_* (**d**), *MTR* (**e**), *MTR_corr_* (**f**), *T1* (**g**), B1 (**h**), and B0map (**i**). The images are displayed with a colormap to facilitate visualization of differences across muscles. The corresponding anatomical image with labeled ROIs is shown in Figure 1B.

Figure 3 and Figure 4 show computed *f* and *MTC* indices at one anatomical level from a young and senior subject, respectively; all images are shown as colormaps with a similar color spectrum. The figures also show the computed *T1*, B1. and B0 maps for the same anatomical slice.

**Figure 5:**
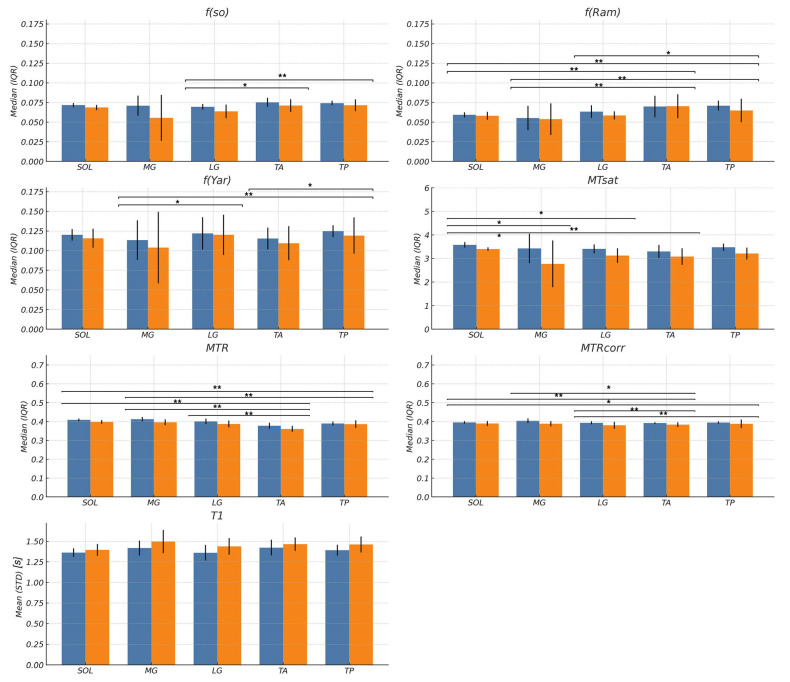
Bar plots of the macromolecular fraction (*f(so)*, *f(Ram)*, *f(Yar)*), qMT indices (*MT_sat_, MTR*, *MTR_corr_*), and T1 are shown for young (blue bars) and old (orange bars) subjects for each muscle region. The significant differences between muscle regions are shown above the horizontal lines with ‘*’ (*p* < 0.05) or ‘**’ (*p* < 0.001). Age-related significant differences were seen in all MT indices and T1 except for *f(Ram)* and *f(Yar)*; Table 2 lists details of the age-related differences. No significant differences in muscle regions were seen in the T1 values.

Figure 5 shows box plots for *f* values from the three qMT sequences: *f(so)*, *f(Ram)*, and *f(Yar)*, MTC indices: *MT_sat_*, *MTR*, and *MTR_corr_*, and *T1* values in ROIs placed in the five muscles segregated by young and senior subjects. Table S1 is a tabular presentation of the box plots. Significant age-related differences were found in *f(so)* and all the MTC indices with lower values of *f, MT_sat_*, *MTR*, and *MTR_corr_* in the senior subjects (Table 2). Surprisingly, *f* extracted from either the five-parameter Ramani or Yarnykh fits did not show significant differences between young and senior subjects even though *f* values were lower in the senior subjects (Table 2). A small, standardized effect size was seen for *f(Ram)* and *f(Yar0*, while *f (so), MTR*, and *MTR_corr_* had a moderate effect, with *MT_sat_* having the highest value in the moderate to large range (Table 2). Significantly longer *T1* values were seen in the senior subjects compared to young subjects. Significant regional differences were found in *f* and in the MTC indices (Figure 5). A common pattern that emerged for regional differences was significant differences between one or more of the plantarflexors and either or both tibialis muscles. Four sequences (*f(so)*, *f(Ram)*, *f(Yar), MTR_corr_)* showed higher values in the tibialis muscles compared to the plantarflexors, while *MT_sat_* and *MTR* showed higher values in the plantarflexors. The results for *MTR* may have been confounded by B1 differences across the muscles. There were no significant regional differences in *T1*.

### 3.3. Correlation of T1 with f and MTC Indices

**Figure 6:**
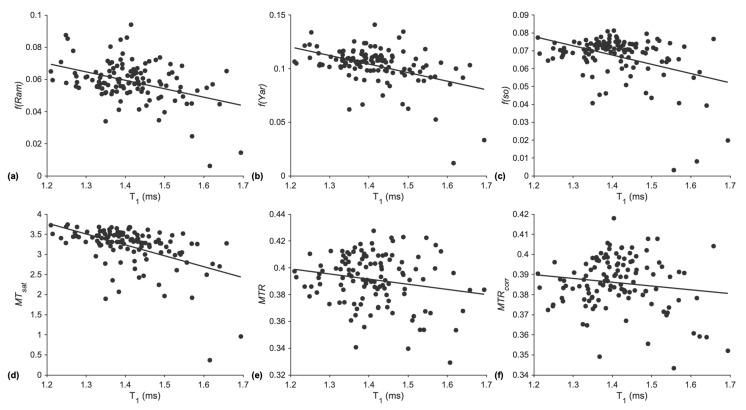
Pearson’s correlation coefficient and *p*-values for each MT sequence are based on the linear fits shown here. *f(Ram)* (r = −0.4, *p* = 5.86 × 10^−6^) (**a**), *f(Yar)* (r = −0.44, *p* = 5.81 × 10^−7^) (**b**), *f(so)* (r = −0.39, *p* = 8.26 × 10^−6^) (**c**), *MT_sat_* (r = −0.51, *p* = 3.49 × 10^−9^) (**d**), *MTR* (r = −0.19, *p* = 0.036 (r = −0.51, *p* = 3.49 × 10^−9^)) (**e**), *MTR_corr_* (r = −0.14, *p* = 0.13) (**f**).

Figure 6 is a plot of the regression lines of *f* or MT index with T1. The points in each plot are the ROI values from the FIVE muscles of each subject including young and senior cohorts. The absolute value of the Pearson correlation coefficient varied from 0.14 (*MTR_corr_*) to 0.51 (*MT_sat_*), and significant correlations with *T1* were seen for the three qMT and *MT_sat_* data while *MTR* and *MTR_corr_* did not yield significant correlations.

## 4. Discussion

The multi-offset and single-offset Ramani model fits yielded macromolecular fractions that were closest to theory in the agarose phantom. The multi-offset Yarnykh model yielded values that were ~40% higher than the theoretical value. However, macromolecular fractions estimated from all three methods were linear with the agarose concentration, showing that these will be good markers of changes in macromolecular fraction that may occur with aging or other musculoskeletal conditions. The other indices of magnetization transfer, *MT_sat_*, *MTR*, and *MTR_corr_*, also exhibit a linear variation with agarose concentration, confirming that they can also be used as biomarkers to track changes in macromolecular fractions even though they are not quantitative. However, it should be noted that of all the indices, MTR has the lowest R^2^ values as well as the highest value of *p* for the fit. This indicates that MTR may not be as reliable a marker to monitor changes/differences as the other indices evaluated here.

The value of *f* from the multi-offset and single-offset Ramani fits for calf muscles in the current study is lower than that reported earlier for calf muscles [9] and for thigh muscles [10]; the *f* value from the multi-offset fit had the bigger discrepancy with earlier reports. Previously reported values of the macromolecular fraction, *f*, ranged from 0.099 (9.9%) in the TA to 0.074 (7.4%) in the LG [9]; the latter study used a multi-offset Ramani model to extract the macromolecular fraction. This is higher than the values of *f* computed in the current study: 0.065 in the TA to 0.060 in the LG for the multi-offset Ramani and 0.075 in the TA to 0.070 in the LG for the single-offset Ramani model. The differences in *f* values are surprising since the current study and that of Sinclair et al. (2010) [9] were similar in terms of the MT RF pulse, MT flip angles, and frequency offsets. The only point of difference was that the current study used fat suppression, in contrast to the earlier work, which did not use any fat suppression. If fat is a significant fraction of the muscle, and if it is unsuppressed, this would imply a lower *f* rather than a higher *f* value since fat exhibits a lower MT effect than muscle. Further, the fat suppression in the current study is accomplished by a water excitation pulse, which has been confirmed from earlier studies to not contribute to incidental magnetization transfer [7]. On the other hand, the multi-offset fit to the Yarnykh model yielded macromolecular fractions higher than obtained from the Ramani model as well from that of earlier studies. Since the Ramani and Yarnykh models differ primarily in the MT RF pulse approximation to the continuous-wave RF, a more detailed analysis of these two approximations is required to understand the differences in the extracted *f* values. *MT_sat_* values seen in the current study were similar to values reported earlier in calf muscles [9]. *MTR* and *MTR_corr_* values reported in the current study are higher than values reported earlier for thigh and calf muscles [16] but this may arise from the fact that the current study employs fat suppression while the earlier study did not include fat suppression. The presence of fat will lead to a decrease in the measured magnetization transfer indices.

This is the first study comparing several magnetization transfer sequences and evaluating their ability to detect age-related differences in macromolecular fraction. All the MT indices except *f* extracted from the multi-offset Ramani and Yarnykh fits yielded significantly lower values in the senior cohort. However, of all the techniques, the multi-offset approach is theoretically the most accurate method to quantify the macromolecular fraction. This holds true when images are acquired with no or very little noise and when there is no motion between the fifteen scans required for quantification. In the current protocol, parallel imaging (acceleration factor of 2) with one average was used for each scan to reduce the total scan time, which in turn leads to noisier images. The multi-offset data is used to fit the two-pool model to multiple parameters, and in the presence of noise, this is very sensitive to fitting errors. In addition, while motion between successive scans was addressed by the affine registration to the reference volume, residual motion mismatch between voxels at different frequency offsets will also contribute to fitting errors. The other techniques are not as impacted by noise as they do not involve multi-parametric fitting and motion between scans is not as pronounced since they involve either two or three acquisitions in contrast to fifteen acquisitions for the multi-offset scan. While these factors may potentially have decreased the sensitivity of the multi-offset scans to detecting age-related changes in macromolecular fraction, it should be acknowledged that there is no reference standard to compare the age-related changes in imaging derived *f* or indices reflective of the macromolecular fraction. This would require histological analysis of biopsy tissue from calf muscles of young and senior subjects to quantify collagen, myofibrillar proteins, and other macromolecules. In the absence of such a reference standard, a definite statement cannot be made that the multi-offset approach is less sensitive to age-related changes; this matter awaits further studies with larger cohort sizes and histological analysis.

Age-related differences in *MT_sat_* between young and senior cohorts reported in the current paper match that reported in an earlier study and had the largest standardized effect size of all the MT indices explored here [7]. The larger effect size implies that *MT_sat_* has a stronger age-related difference relative to its variability. Age-related differences in *MTR_corr_* seen in the current paper are like that reported earlier; however, the % change with age was marginally higher in the earlier paper [16]. These differences with age may be attributed to the fact that the paper by Morrow et al. (2014) [16] did not use fat suppression. As the fat fraction increases with age, the prior paper would have measured the combined effects of magnetization transfer and fat infiltration. Jerban et al. (2024) [27] used MT modeling with ultrashort echo time acquisition (UTE-MT) to study age-related differences in skeletal muscle. The latter paper found a large change in *f* with age (~19% change) when comparing young to senior cohorts of all-female subjects. This is much larger than the age-related differences found in the current paper (~4.2% in *f(so)*) and by Morrow et al. (2014) [16] (~3.4% in *MTR_corr_*). Some of these differences may arise from the fact that the UTE-MT study included only female subjects. Gender-related differences in *MT_sat_* have been reported earlier, and it is possible that there are also gender-specific age-related changes [18]. Other reasons for the discrepancy in the current study and the UTE-MT may arise from (i) the fact that the mean age of the senior cohort in the current study was 68 years while that in the UTE-MT study was 75 years, (ii) that fat was not suppressed in the UTE-MT study and could also have contributed to an additional decrease in macromolecular fraction with age, and (iii) the primary reason for the discrepancies in the size effect is most likely due to the different sequences used. The UTE-MT sequence can capture the signal from very low T2 species, and thus, it is likely that additional MT effects in low T2 species are also included in UTE-MT compared to the longer TE sequences used in the current study. It would be interesting to map *f* using MT sequences at increasing TEs from ultrashort to longer TEs to verify if there is a change in *f* with TE.

Earlier studies on muscle MTC imaging hypothesized that the macromolecule responsible for the observed MT effect was the collagen of the extracellular matrix [9,10]. However, more recent studies exploring age-related changes in MTC indicate that both myofibrillar proteins and collagen may be the potential macromolecules that contribute to the MTC effect [16,27]. Support that myofibrillar proteins contribute to the MT effect also comes from a rat model study where *MTR* was used to track fiber formation after injection of human muscle progenitor cells for development of muscle tissue [14]. *MTR* increased with myogenesis and correlated well with muscle contractility measurements. The authors of the latter paper [14] have advanced the hypothesis that higher MT in muscle may arise from a large abundance of macromolecules in the form of aligned muscle fibers in well-developed muscle tissue. A study exploring age-related changes in *MT_sat_* reported a significant decrease in MTC in all the calf muscles [7]. The study by White et al. (2022) [7] concluded that the MT decrease with age may arise primarily from loss of myofibrillar proteins rather than increase in collagen. Another study of age-related MTC effects in muscle used *MTR_corr_* and reported a significant decline of *MTR* with age in the calf muscles [16]. The latter authors also suggest an *MTR* age-dependence independent of age-related muscle lipid increases, presumably reflecting myofiber quality and density changes. It is quite likely that age-related changes in both myofibrillar proteins as well as in collagen contribute to the observed age-related decrease in magnetization transfer effect. It should be noted that myofibrillar proteins impact force production while collagen affects force transmission. Thus, measurement of the macromolecular fraction or a surrogate index will allow an evaluation of muscle quality.

It should be noted that other neuromuscular conditions can result in changes that occur predominantly in collagen as well. Using animal models, a recent study identified that the dominant change in pathologic rotator cuff muscle is the increase in collagen content aside from the fatty infiltration [28]. This increase in collagen content (quantified by connective tissue fraction from histological analysis) correlated with the increase in macromolecular fraction measured by UTE-MT imaging with fat suppression. The latter study also emphasized the need for fat suppression to remove the confounding effects of fat infiltration on MTC indices. The studies on aging and injury model clearly indicate that it is important to correlate MT studies with histological analysis as different macromolecules (myofibrillar proteins and/or collagen) may be affected to varying extents depending on the specific muscle pathology (e.g., aging, injury). The correlation of the macromolecular fraction in aging muscle from qMT/MTC imaging to macromolecular pool(s) from histological analysis has not yet been established. In contrast, in the brain, it has been established that the macromolecular fraction from qMT has a high degree of correlation with the myelin content [29].

Li et al. determined the pool size ratio, *F*, in normal thigh muscle as well as in thigh muscle of subjects with polymyositis—the latter is an inflammatory disease that causes muscle weakness [10]. They found that *F* was significantly reduced in all the thigh muscles, and they attributed this reduction to the presence of inflammation (edema has negligible magnetization transfer effects). While inflammation is not as severe with age as it is in polymyositis, earlier studies clearly revealed that *T1* was prolonged with age, potentially indicating a degree of age-related inflammatory processes [7]. It is then likely that the observed lower MT values with age may reflect differences in inflammation rather than differences in the macromolecular fraction. Or it could be the combined effect of age-related differences in the macromolecular fraction and inflammation. To test this, the Pearson correlation coefficient between each MT index and *T1* was determined. The coefficients were low to moderate, but all correlations were significant (*p* < 0.001). This indicates that while age-related increase in inflammation (resulting in increased *T1*) may contribute to a decrease in the magnetization transfer effect, there are also other physiological variables independent of inflammation that contribute to the MT process. A multi-parametric MR study has shown that of all the MR parameters studied (fat fraction, *T1*, T2, and magnetization transfer), MT had the largest effect size with age [16]. Thus, monitoring MT effects will prove to be an effective biomarker of age-related differences in skeletal muscle.

While, in the above discussion, the macromolecular fraction has been considered the primary source for MTC, there are other tissue factors that may contribute to the observed MTC differences between the young and senior cohorts. Magnetization transfer contrast is based on interactions between the macromolecular and water protons in the hydration layers [30]. Skeletal muscle hydration is known to decrease with age, and this may potentially lead to a decrease in MTC as well [31]. However, it should be noted that unlike other MR parameters, MTC is orientation-independent, making it a desirable biomarker. MR spectroscopy of muscle metabolites showed that while water T_2_ relaxation times depend on muscle fiber orientation relative to the main magnetic field, magnetization transfer rates are independent of fiber orientation [32]. Further, while there is the large orientational dependence of T2 relaxation time in anisotropic, collagen-rich tissues, magnetization transfer contrast is relatively independent of the tissue orientation in the main magnetic field [33]. Thus, age-related differences in fiber orientation and or collagen anisotropy may not contribute to the observed MTC differences between the young and senior cohorts. The contribution of inflammation, reflected in prolonged T1 relaxation values, was factored in to explain some of the age-related differences in MTC, but it should be acknowledged that there may be contributions to MTC from sub-clinical inflammation not captured in the measured T1 values. Future studies based on histological analysis of muscle biopsy samples will be necessary to identify the source of MTC in skeletal muscle.

When comparing the different sequences, the multi-offset sequence had the longest acquisition times and complex processing. Further, it did not show significant changes in *f* with age. The single-offset model is based on a much shorter acquisition time, does not require fitting to a model, and further, revealed significant changes with age. However, there are some model parameters that are kept fixed to derive the simpler model [10]. While these parameters have been shown to remain unchanged across muscles in normal subjects, there is a possibility that these may change with neuromuscular disease condition. Of the other sequences, *MTR_corr_* corrects for B1 inhomogeneities but it requires a large region of muscle that can be used to generate the correction factor and may not work when fat/fibrosis fractions are high. Of all the indices tested, *MT_sat_* appears as the most clinically relevant biomarker of aging in terms of acquisition speed and computational simplicity. Further, *MT_sat_* is semi-quantitative, and unlike *MTR*, it is relatively insensitive to B1 inhomogeneities and *T1*. The *MT_sat_* technique also does not require any special sequences and can be implemented with routinely available spoiled gradient echo sequences. Further, *MT_sat_* also had the highest size effect when comparing the young and senior cohorts.

A key limitation of this study is the small sample size (15 young and 9 senior), which may potentially have limited the ability to detect age-related changes in the macromolecular fraction computed from the multi-offset fits to the two-pool model. However, the sample size was sufficient to detect age-related changes in *f(os)*, *MT_sat_*, *MTR*, and *MTR_corr_*. While the current study showed that macromolecular fractions differ with age, it cannot identify the changes in different macromolecules that resulted in the observed decrease. Addition of histological analysis would have provided this correlation but was outside the scope of the current study. However, it should be noted that the current study included fat suppression, which served to disambiguate the confounding effects of increased fat infiltration with age. Further, correlation analysis to *T1* showed that MT indices reflect more than just changes in inflammation. Future studies with larger cohort sizes that include histological analysis may be able to identify age-related differences in macromolecules, hydration, fat fraction, and inflammation and how the age-related differences in these factors are reflected in the MT indices.

## 5. Conclusions

Different MRI fat-suppressed-based approaches to identifying age-related differences in magnetization transfer effects in muscle were evaluated. The multi-offset approaches (Ramani and Yarnykh) based on fitting five model parameters did not identify significant differences in macromolecular fraction between young and senior cohorts. The accelerated single-offset model that computes the macromolecular fraction by fixing the values of three of the model parameters showed a significantly lower macromolecular fraction in the senior cohort. The other three indices of magnetization transfer also showed significant age-related changes, with lower values of the MT index in the senior cohorts. The Pearson correlation coefficient between each MT index and T1 identified low to moderate but significant correlations (*p* < 0.001). This indicates that there are other physiological variables, independent of inflammation, that contribute to the MT process. This study identified *MT_sat_* as having the best potential for future clinical applications to characterize/monitor neuromuscular diseases, as the approach is fast, requires relatively easy computations, and has demonstrated sensitivity to detect age-related changes in macromolecular fractions.

## Figures and Tables

**Table 1:** *f* values extracted from the 5-parameter and 2-parameter fits to the 2-pool model.

Agarose Concentration (%)	0.5	1	2	3	4
Theory *	0.002	0.004	0.009	0.013	0.017
*f(Ram)*	0.003	0.005	0.008	0.012	0.016
*f(Yar)*	0.004	0.007	0.013	0.02	0.024
*f(so)*	0.001	0.004	0.008	0.014	0.017

* Macromolecular fraction calculated for each agarose concentration as the fraction of protons in agarose to the total protons in the phantom solution.

**Table 2:** Summary statistics of age-related differences in *f* and MT indices.

MT	*p* (2-Sided)	^1^ HL (Y−O)	^2^ 95% CI L	^2^ 95% CI H	^3^ r	^4^ Magnitude	^5^ Dir
*f(Ram)*	0.076	0.004	0.0	0.008	0.16	small	Y > O
*f(Yar)*	0.067	0.005	0.0	0.01	0.17	small	Y > O
*f(so)*	<0.001	0.006	0.003	0.008	0.42	medium	Y > O
*MT_sat_*	<0.001	0.276	0.183	0.368	0.49	medium	Y > O
*MTR*	<0.001	0.016	0.009	0.022	0.38	medium	Y > O
*MTR_corr_*	<0.001	0.012	0.007	0.017	0.44	medium	Y > O

^1^ HL: Hodges–Lehmann estimate of the age difference (young–old), ^2^ 95% CI L and 95% CI H are the low and high values, respectively, of the 95% confidence interval (CI), ^3^ r: rank-biserial correlation is the standardized effect size, ^4^ magnitude is the qualitative descriptor of the standardized effect size, ^5^ Dir is the direction of the values (young is always higher than old for all indices).

## Data Availability

The raw data supporting the conclusions of this article will be made available by the authors on request.

## Supplementary Materials

The following supporting information can be downloaded at https://www.mdpi.com/article/10.3390/tomography11090103/s1, Table S1: Magnetization transfer (MT) indices and T1 for the calf muscles for the young (Y) and senior (S) cohorts.
