# Supplementary material for: Evaluation of Magnetization Transfer Contrast Sequences: Application to Monitor Age-Related Differences in Muscle Macromolecular Fraction
Source: Tomography. 2025 Sep 5;11(9):103. doi: 10.3390/tomography11090103 (PMC12473340; doi:10.3390/tomography11090103)
Supplement: Supplementary file 1 [file tomography-11-00103-s001.zip › tomography-3788210-supplementary.pdf]

Table S1. Magnetization Transfer (MT) indices and T1 for the calf muscles for the young (Y) and senior (S) cohorts.

MT/T1	C	SOL	MG	LG	TA	TP
$f(Ram)^{2,3,4,5,6}$	Y	0.056(0.003)	0.052(0.015)	0.060(0.008)	0.065(0.013)	0.066(0.006)
	S	0.055(0.006)	0.051(0.026)	0.055(0.008)	0.066(0.014)	0.061(0.016)
$f(Yar)^{4,7,8}$	Y	0.107(0.007)	0.102(0.023)	0.109(0.017)	0.103(0.012)	0.111(0.007)
	S	0.104(0.010)	0.094(0.047)	0.107(0.021)	0.099(0.020)	0.106(0.020)
$f(so)^{*,1,2}$	Y	0.072(0.003)	0.071(0.014)	0.070(0.004)	0.075(0.007)	0.074(0.004)
	S	0.069(0.004)	0.055(0.035)	0.064(0.012)	0.071(0.011)	0.071(0.010)
$MT_{sat}^{*,5,9,10}$	Y	3.577(0.130)	3.426(0.657)	3.405(0.210)	3.297(0.361)	3.474(0.158)
	S	3.396(0.125)	2.771(1.317)	3.118(0.352)	3.078(0.449)	3.209(0.334)
$MTR^{*,1,3,4,5,6}$	Y	0.409(0.007)	0.412(0.013)	0.401(0.016)	0.377(0.018)	0.390(0.012)
	S	0.398 (0.011)	0.396(0.018)	0.387(0.022)	0.361(0.019)	0.386(0.025)
$MTR_{corr}^{*,1,2,3,5,6}$	Y	0.384(0.012)	0.391(0.008)	0.383(0.006)	0.401(0.008)	0.395(0.008)
	S	0.378(0.009)	0.381(0.011)	0.367(0.017)	0.386(0.025)	0.389(0.023)
$T1^{*}$(s)	Y	1.377(0.056)	1.412(0.148)	1.395(0.158)	1.417(0.075)	1.393(0.065)
	S	1.381(0.106)	1.486(0.275)	1.435(0.208)	1.460(0.133)	1.451(0.155)

C: Cohort; Median and Interquartile Range (in brackets) for all MT indices; Mean and Standard Deviation (in brackets) for T1 (seconds); *: significant age-related differences, young greater than senior; 1: significant differences in LG and TA; 2: significant differences in LG and TP ; 3: significant differences in MG and TA ; 4: significant differences in MG and TP; 5: significant differences in SOL and TA; 6: significant differences in SOL and TP; 7: significant differences in MG and LG; 8: significant differences in TA and TP; 9: significant differences in MG and SOL; 10: significant differences in LG and SOL.
